# Reference intervals for thyroid disorders calculated by indirect method and comparison with reference change values

**DOI:** 10.11613/BM.2023.010704

**Published:** 2022-12-15

**Authors:** Zeynep Yildiz, Lale Köroğlu Dağdelen

**Affiliations:** Department of Biochemistry Laboratory, Dr Lutfi Kirdar City Hospital, Istanbul, Turkey

**Keywords:** reference interval, thyroid hormone, indirect method, reference change value

## Abstract

**Introduction:**

The aim of the study was to calculate reference intervals (RIs) for thyroid stimulating hormone (TSH), free thyroxine (fT4) and free triiodothyronine (fT3) and evaluate the clinical significance of these intervals by use of reference change values (RCV) of the analytes.

**Materials and methods:**

Laboratory patient data between August and December 2021 were evaluated for the study. A total of 188,912 patients with TSH, fT4, fT3, anti-thyroid peroxidase antibodies (Anti-TPO) and anti-thyroglobulin antibodies (Anti-Tg) results were evaluated. All measurements were performed on Cobas c801 (Roche Diagnostics, Penzberg, Germany) using electrochemiluminescence immunoassay technology. Estimated RIs were compared with manufacturer’s by means of RCVs of analytes.

**Results:**

Thyroid stimulating hormone values didn’t differ significantly by gender and age. The combined RIs for whole group (N = 28,437) was found as 0.41-4.37 mIU/mL. Free T4 values (11.6-20.1 pmol/L, N = 13,479 in male; 10.5-19.5 pmol/L, N = 17,634 female) and fT3 values (3.38-6.35 pmol/L, N = 2,516 in male; 3.39-5.99 pmol/L, N = 3,348 pmol/L in female) significantly differed by gender (P < 0.050). Both fT4 and fT3 values also showed significant differences in age subgroups comparisons. So, male and female RIs were represented separately for age subgroups. When compared with manufacturer’s RIs, TSH whole group and fT4 subgroups RIs didn’t exceed the analytes’ RCVs, but this difference was greater for fT3.

**Conclusions:**

Reference interval estimation by use of indirect method out of laboratory data may be more accurate than manufacturer provided RIs. This population based RIs evaluated using RCV of analytes may provide useful information in clinical interpretation of laboratory results.

## Introduction

The reference interval (RI) is defined as the interval corresponding to the central 95% of values of a reference population, including the two boundary limits: upper reference limit (URL) and lower reference limit (LRL). This interval is supposed to represent a well-defined status of physiological conditions, mainly “goodhealth” together with other analytic variations of the assay system and biologic variations of the analyte in the particular population (*1*, *2*). Thus, it is recommended that medical laboratories determine their own RIs to cover the variability of their local populations and their specific analytic methods and devices. For the process of RI determination, the Clinical Laboratory Standards Institute (CLSI) recommends “direct” approach, where well defined reference subjects are selected with pre-defined criteria and the measurements are done afterwards (*3*). Direct method is hard to apply for every laboratory in routine practice for it demands much time and money (*4*). The alternative approach is the “indirect” method where test results of patients that were ordered for screening, diagnosis or follow-up purposes, are derived from laboratory information system (LIS) and used to determine the RIs. Indirect method generally uses the data of outpatients and primary care patients and exclude the results that don’t fit the general distribution of data. This method is faster and cheaper; it doesn’t cause discomfort or any additional risk to patients, nor any additional workload to laboratory staff (*5*). Besides, the results obtained by the indirect method are closer to the actual state of the population of a given region, because they take into account the analytical and biological variability of the analysed parameter (*1*). Recently, The International Federation of Clinical Chemistry and Laboratory Medicine (IFCC) Committee on Reference Intervals and Decision Limits encourages the use of indirect methods to establish and verify reference intervals (*2*).

The prevalence of subclinical hypothyroidism is very high; up to 4.8% in Europe in a recent meta-analysis (*6*). Subclinical hypo/hypertiroidism is diagnosed in the laboratory, so the need for accurate both upper and lower reference limits have been strongly emphasized. But there are still discrepancies between RIs used in laboratories as well as up-to-date scientific literature (*1*). In recent studies, calculated thyroid stimulating hormone (TSH) upper limits varied between 5.28 to 2.84 mIU/L and lower limits from 0.17 to 0.64 mIU/L with remarkable differences in RIs of free triiodothyronine (fT3) and free thyroxine (fT4) too (*7*-*10*). Variations in the results of thyroid function tests in a healthy population may be due to analytical (CV_A_), intra-individual (CV_İ_) and inter-individual (CV_G_) variations. Thyroid stimulating hormone, in its nature, has various isoforms with different glycosylation patterns in circulation. Glycosylation may alter the biological activity of the hormone but the immunological pattern is not affected causing a normal result when measured with an immunoassay (*11*). Moreover, this heterogeneity may induce problems in the standardization of TSH measurements, which may explain differences of around 30-40% in TSH values due to assay technology (*12*). Besides, thyroid hormones, especially TSH, as well as fT4 and fT3, show a large biological variation, mainly CV_G_. In such a situation, it is not advisable to use other populations’ RIs; at least each laboratory should establish its own population-based intervals by means of a cheap and easy optimized indirect statistical method out of a large data set. For any analyte with a great CV_G_ like TSH, there is a need to have more granularity in the RI by partitioning into more homogenous subgroups by age and/or gender *etc.* (*13*).

Reference change value (RCV) is the critical difference that may be attributed to a real clinical change, which depends mainly on the CV_A_ and CV_İ_ variations of the particular analyte (*14*). Assuming CV_A_ is often very small for most assays, RCV largely depends on the CV_İ_ of the analyte (*15*). This RCV concept will offer clinician a more accurate tool to detect changes in a patient’ health status. Furthermoore, this is surely a better approach than the population-based RIs because many analytes have been shown to have a great CV_İ_ (*14*). Biological variation estimates of many analytes are available at www.biologicalvariation.eu (*16*).

In this study, we calculated our age and gender specific RIs for TSH, fT4 and fT3 on a large data. We compared our results with those of manufacturers and others in medical literature and tried to establish the significance of variations in terms of RCVs attributed to these 3 analytes to contribute their clinical interpretation.

## Materials and methods

### Study design

This is an indirect method of reference value analysis using laboratory patient data of Kartal Dr Lütfi Kırdar City Hospital localized in Anatolian region of Istanbul which gives healthcare with 1205 inpatient beds and 10 thousand daily outpatient visits. Besides our hospital, our core laboratory accepts approximately 15 thousand daily samples from 8 other hospitals and 166 primary care centers serving a large population living in both eastern, mostly, and western regions of the city.

### Subjects

**Figure 1:**
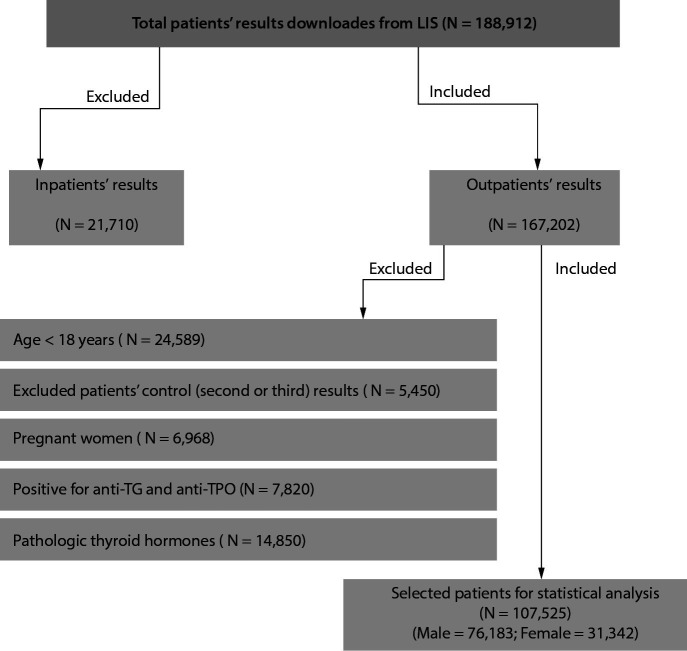
Flow diagram showing the selection criteria for patients’ results for the estimation of reference intervals of thyroid parameters using the indirect method. LIS – laboratory information system. Anti-TPO - anti-thyroid peroxidase antibodies. Anti-Tg - anti-thyroglobulin antibodies.

Laboratory information system patient data between August and December 2021 were evaluated for the study. A total of 188,912 patients having all of TSH, fT4, fT3, anti-thyroid peroxidase antibodies (Anti-TPO) and anti-thyroglobulin antibodies (Anti-Tg) results were downloaded from our LIS. Only the first result of each patient was included. Patients < 18 years of age, pregnant females, inpatients results and patients with pathologic fT4, fT3, Anti-Tg, Anti-TPO results and/or with TSH > 10 mIU/mL were excluded. The flow diagram of study is shown in Figure 1. Finally 107,525 (76,183 male and 31,342 female) were included for statistical analysis. Then 103 values for TSH, 66 values for fT4 and 153 values for fT3 were detected as outliers and excluded. All data were grouped by gender and each gender by age decades as 18-30, 31-40, 41-50, 51-60, 61-70 and > 71 years subgroups.

### Blood sampling

Only morning fasting samples were accepted for routine chemistry analyses. Blood was drawn into BD Vacutainer® SST™II tubes (Becton Dickinson Italia S.p.A., Milan, Italy, ref. n. 366566) with serum separator in all centers and was centrifuged at 3000xg for 10 minutes and then transported to our core laboratory at 0-5 °C and were measured within 2 hours of admittance.

This study, was approved by our institution’s Ethical Committee (İstanbul, Kartal Dr Lütfi Kırdar City Hospital Ethical Committee; 22.06.2021/ 2021/514/204/2).

### Methods

Roche TSH, fT4, fT3, Anti-TPO and Anti-Tg assays are based on electrochemiluminescence immunoassay to be used on Cobas e 801 immunoanalyser c801 (Roche Diagnostics, Penzberg, Germany). The TSH test method is sandwich immunoassay, while the others are competetive immunoassays. The TSH assay is calibrated against 2. International Reference Preparation (IRP) WHO Reference Standart 80/558, while fT4 assay was calibrated against the Enzymun-Test which had been calibrated against an equilibrium dialysis fT4 analysis of Roche. The fT3 assay was calibrated against an equilibrium dialysis fT3 analysis at Roche. Thyroid stimulating hormone assay has a functional sensitivity of < 0.005 mIU/L with a manufacturer provided RI of 0.27-4.20 mIU/L. Free thyroxine assay has a limit of detection (LOD) of 0.5 pmol/L with a manufacturer provided RI of 12-22 pmol/L. Free triiodothyronine assay has a LOD 0.6 pmol/L with a manufacturer provided RI of 3.1-6.8 pmol/L. Detection limits and RIs for anti-Tg assay was 7.16 and < 115 IU/mL and for anti-TPO was 9 and < 34 IU/mL. Both assays were calibrated against National Institute of Biological Standarts and Controls materials (65/93) and (66/387), respectively. Two levels of commercial control sera provided by the manufacturer were conducted daily to ensure internal quality control.

### Statistical analysis

For each analyte, male and female frequency distributions were evaluated separately. Shapiro-Wilk test was used to assess whether the distribution of data was Gaussian. Logaritmic transformations were done. Outliers were tested using Tukey’s method and subsequently eliminated. Age partitioning were done as decades according to existing medical literature (*17*, *18*). Reference intervals were derived by non-parametric method and reported as the 2.5 and 97.5 percentiles with 90% confidence intervals for lower and upper limits. The significance of differences between gender and age subgroups were assessed by the standard normal deviation test (Z-test) and RIs were reorganized (*14*). The reference change values were used for comparison of the calculated subgroup RIs with RIs provided by the manufacturer. If the calculated % difference was less than the RCV, the difference was not significant (*14*, *19*). Reference change values were calculated as described by Fraser *et al.* (*20*). CV_A_s were calculated out of laboratory internal quality control data with two levels control sera measurements for 20 days and calculated with the formula Total CV = √(CV of Level 1)^2^+(CV of Level 2)^2^. CV_İ_s of analytes were taken from EFLM database (*16*).

## Results

Analytical CV_A_s were found as 7.20% for TSH, 5.63% for fT4, 4.61% for fT3, RCVs for 3 analytes were found as 53.0% for TSH, 20.7% for fT4 and 18.9% for fT3 (Supplemental Table 1).

**Figure 2:**
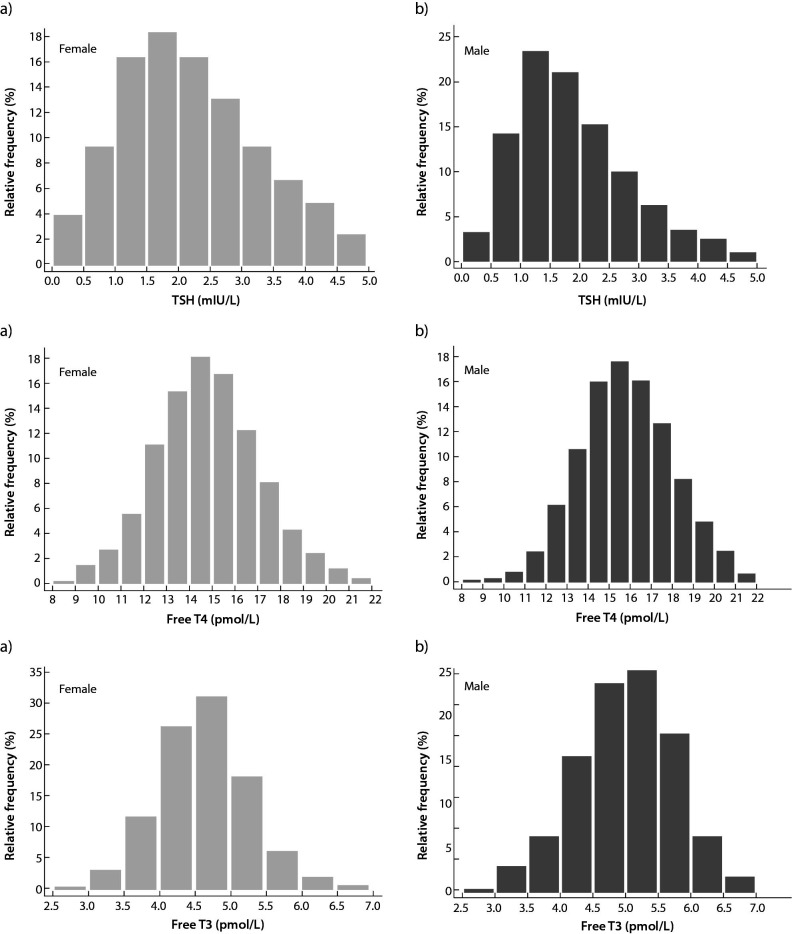
Frequency distribution of thyroid hormones in a) females; and b) males. TSH - thyroid stimulating hormone. fT4 - free thyroxine. fT3 - free triiodothyronine.

Frequency distributions of all 3 analytes were non-Gaussian for both genders initially, especially male and female TSH values were quite positively skewed. After logarithmic transformations and exclusion of outliers, distributions turned to be Gaussian for all analytes except for slightly skewed TSH male values with a longer tail towards higher values. Approximately 90% of male had TSH values < 3.5 mIU/L. For fT4 and fT3, central 50% distribution values of male were higher than female. Four percent of male but 10% of female had fT4 values < 12 pmol/L, while 10% of male but 4% of female had fT3 values > 6 pmol/L. Frequency distribution diagrams of 3 analytes for male and female are shown in Figure 2.

Age and gender-specific descriptive statistical data of subgroups are presented in Table 1. Higher TSH values were observed in females in total and all age subgroups, but neither difference was statistically significant (P > 0.050). Mean values of both male and female TSH decreased by age. Age group 18-30 had the highest values as > 70 years group had the lowest. No age related significant difference was found. Thus, TSH RIs, independent of age and gender, are represented as whole group: 0.41-4.37 mIU/L.

**Table 1:** Age and gender-specific descriptive statistical data of subgroups

	**Males**		**Females**	
**Age (years)**	**Mean**	**SD**	**Mean**	**SD**
**TSH (mIU/L)**				
**18-30**	2.09	1.94	2.26	1.02
**31-40**	1.86	0.90	2.17	1.05
**41-50**	1.81	0.93	2.18	1.03
**51-60**	1.83	0.95	2.16	1.12
**61-70**	1.82	0.97	2.12	1.09
**> 70**	1.83	1.01	2.02	1.14
**All**	1.87	0.95	2.18	1.07
**fT4 (pmol/L)**				
**18-30**	16.48	2.12	14.46	2.19
**31-40**	15.93	2.12	14.36	2.20
**41-50**	15.66	2.13	14.75	2.13
**51-60**	15.61	2.10	15.24	2.18
**61-70**	15.46	2.19	15.53	2.23
**> 70**	15.40	2.30	15.58	2.33
**All**	15.75	2.18	14.78	2.24
**fT3 (pmol/L)**				
**18-30**	5.55	0.60	4.97	0.61
**31-40**	5.29	0.63	4.77	0.63
**41-50**	5.17	0.62	4.60	0.56
**51-60**	4.86	0.63	4.59	0.56
**61-70**	4.64	0.61	4.39	0.60
**> 70**	4.22	0.65	4.13	0.63
**All**	4.99	0.74	4.64	0.64
TSH - thyroid stimulating hormone. fT4 - free thyroxine. fT3 - free triiodothyronine.				

Free T4 and fT3 values showed age and gender specific differences, so RIs were represented separately (calculated Z/critical Z values were seen on Supplemental Tables 2, 3 and 4). The 2.5 and 97.5 percentiles derived by non-parametric method and the significance of partitioning were presented in Table 2.

**Table 2:** TSH, fT4 and fT3 reference intervals (2.5 and 97.5 percentiles) derived by non-parametric method with 90% confidence intervals for lower and upper limits

		**Males**			**Females**		
	**Age (years)**	**N**	**Lower limit** **(90% CI)**	**Upper limit ** **(90% CI)**	**N**	**Lower limit** **(90% CI)**	**Upper limit** **(90% CI)**
**TSH (mIU/L)**	**18- 30**	2198	0.69 (0.65-0.73)	4.29 (4.20-4.37)	3962	0.55 (0.51-0.62)	4.47 (4.42-4.53)
	**31-40**	1929	0.52 (0.49-0.57)	4.12 (4.05-4.22)	3282	0.40 (0.34-0.45)	4.45 (4.42-4.51)
	**41-50**	2643	0.48 (0.43-0.51)	4.15 (3.99-4.26)	2822	0.29 (0.22-0.36)	4.46 (4.39-4.52)
	**51-60**	2930	0.42 (0.38-0.46)	4.17 (4.07-4.24)	2290	0.26 (0.20-0.31)	4.48 (4.38-4.55)
	**61-70**	2436	0.37 (0.33-0.41)	4.14 (4.04-4.23)	1581	0.26 (0.22-0.38)	4.43 (4.31-4.50)
	**>70**	1399	0.31 (0.25-0.35)	4.29 (4.17-4.41)	965	0.22 (0.17-0.32)	4.50 (4.38-4.62)
	**All**	13535	0.45 (0.43-0.47)	4.19 (4.15-4.23)	14902	0.36 (0.33-0.39)	4.46 (4.44-4.49)
**fT4 (pmol/L)**	**18- 30**	2153	12.1 (11.9-12.4)	20.4 (20.3-20.5)	4895	10.1 (10.0-10.3)	18.9 (18.8-19.1)
	**31-40**	1989	11.9 (11.7-12.1)	20.3 (20.1-20.4)	4140	10.1 (9.98-10.2)	18.7 (18.6-18.9)
	**41-50**	2551	11.7 (11.6-11.9)	19.9 (19.7-20.1)	3269	10.9 (10.7-11.0)	19.5 (19.4-19.7)
	**51-60**	2825	11.5 (11.4-11.7)	20.0 (19.7-20.1)	2490	11.1 (10.9-11.3)	19.9 (19.7-20.1)
	**61-70**	2498	11.4 (11.2-11.6)	20.1 (19.7-20.3)	1747	11.5 (11.2-11.7)	20.3 (20.0-20.5)
	**>70**	1463	11.06 (10.9-11.3)	20.04 (19.8-20.3)	1093	11.0 (10.7-11.3)	20.2 (20.0-20.4)
	**All**	13479	11.6 (11.5-11.7)	20.1 (20.1-20.2)	17634	10.5 (10.4-10.5)	19.5 (19.4-19.6)
**fT3 (pmol/L)**	**18- 30**	413	4.14 (4.03-4.34)	6.64 (6.55-6.66)	709	3.75 (3.60-3.92)	6.32 (6.16-6.41)
	**31-40**	364	3.91 (3.16-4.17)	6.50 (6.31-6.58)	594	3.61 (3.56-3.75)	6.10 (6.02-6.35)
	**41-50**	493	3.79 (3.54-3.95)	6.34 (6.22-6.52)	671	3.51 (3.44-3.57)	5.69 (5.63-5.75)
	**51-60**	534	3.62 (3.54-3.70)	6.09 (6.01-6.17)	614	3.48 (3.42-3.55)	5.69 (5.63-5.76)
	**61-70**	445	3.29 (3.12-3.45)	5.70 (5.64-5.81)	446	3.34 (3.16-3.40)	5.83 (5.49-6.02)
	**> 70**	267	2.96 (2.84-3.07)	5.49 (5.37-5.62)	314	2.90 (2.80-3.00)	5.37 (5.27-5.47)
	**All**	2516	3.38 (3.28-3.45)	6.35 (6.31-6.45)	3348	3.39 (3.34-3.43)	5.99 (5.91-6.06)
CI - confidence interval. Lower Limit - 2.5 percentile. Upper limit - 97.5 percentile. TSH - thyroid stimulating hormone. fT4 - free thyroxine. fT3 - free triiodothyronine.							

Percent difference for LRLs and URLs between RIs calculated in this study and manufacturer provided were smaller than corresponding RCVs for TSH and fT4 but not for fT3 (Table 3). So manufacturer provided RIs were clinically different from our population based RIs for fT3, but not for fT4 and TSH.

**Table 3:** Comparison of estimated reference intervals with reference intervals provided by the manufacturer.

	Males		Females		
Age (years)	**LRL** (% difference)*		**URL** (% difference)*		**RCV**
	**TSH** (0.27–4.20 mIU/L)^†^				
All^§^	0.41 (+ 34.4)		4.37 (+ 3.89)		53
**fT4** (12–22 pmol/L)^†^					
18-30	12.1 (+ 0.9)	20.4 (- 7.8)	10.1 (- 18.8)	18.9 (- 16.4)	
31-40	11.9 (- 0.8)	20.3 (- 8.4)	10.1 (- 18.8)	18.7 (- 17.6)	
41-50	11.7 (- 2.6)	19.9 (- 10.6)	10.9 (- 10.1)	19.5 (- 12.8)	
51-60	11.5 (- 4.3)	20 (- 10.0)	11.1 (- 8.1)	19.9 (- 10.6)	20.7
61-70	11.4 (- 5.3)	20.1 (- 9.5)	11.5 (- 4.3)	20.3 (- 8.4)	
>70	11.06 (- 8.5)	20.04 (- 9.8)	11.0 (- 9.1)	20.2 (- 8.9)	
**fT3 (3.1–6.8 pmol/L)^†^**					
18-30	4.14 (+ 25.1)^ǂ^	6.64 (- 2.4)	3.75 (+ 17.3)	6.32 (- 7.6)	
31-40	3.91 (+ 20.7)^ǂ^	6.50 (- 4.6)	3.61 (+ 14.1)	6.10 (- 11.5)	
41-50	3.79 (+ 18.2)	6.34 (- 7.3)	3.51 (+ 11.7)	5.69 (- 19.5)^ǂ^	
51-60	3.62 (+ 14.4)	6.09 (- 11.7)	3.48 (+ 10.9)	5.59 (- 21.6)^ǂ^	18.9
61-70	3.29 (+ 5.8)	5.70 (- 19.3)^ǂ^	3.34 (+ 7.2)	5.38 (- 26.4)^ǂ^	
> 70	2.96 (- 4.72)	5.49 (- 23.4)^ǂ^	2.90 (- 6.9)	5.37 (- 26.6)^ǂ^	
*Percent differences between group and manufacturer LRLs and URLs are separately calculated and compared with RCVs of the corresponding analytes. ^†^Manufacturer recommended reference range. ^ǂ^Percent differences greater than the RCV of the analyte. ^§^TSH combined RIs for whole group. LRL - lower reference limit. URL - upper reference limit. TSH - thyroid stimulating hormone. fT4 - free thyroxine. fT3 - free triiodothyronine.					

## Discussion

In this study we calculated population based RIs for TSH, fT4 and fT3 out of our hospital data by use of indirect method. The RIs of individual groups found in this study were in accordance with manufacturer provided values for TSH and fT4 but not for fT3 when compared with their corresponding RCVs. Subclinical hypothyroidism has a high prevalence all over the world and diagnosis is made mainly by laboratory tests (*5*, *6*). Depending on its importance, there are many reports about RIs of TSH, and also a few for fT4 and fT3 in the medical literature. Table 4 shows the variabilities of studies in literature: different analysers, different populations, study type, statistical calculations (21, 22). As a result, LRLs of the studies vary between 0.17-0.75 mIU/L and URLs between 2.84-5.32 mIU/L for TSH; even 4 studies on the same platform (Roche) like ours, LRLs and URLs vary between 0.43-0.75 and 3.93-5.32 mIU/L, respectively (*5*, *7*, *8*, *18*). Our TSH RI was found in the middle of this range. Discrepancies were also observed when studies were grouped as direct and indirect RI calculation methods. LRLs were 0.56 and 0.75 mIU/L; URLs were 4.45 and 5.32 mIU/L with two direct methods on the same analyser (*7*, *8*). Thus, differences between RIs in the medical studies were hard to attribute to any variable. In our study we used an indirect method using our hospital’s patient data, selecting outpatients and primary care patients, whom these tests were probably ordered for screening (*23*). In two of the studies gender related RIs were established and femaleTSH RIs had higher values compared to male (*9*, *10*). In our study TSH values of female were higher too, but not statistically significant. Also, male fT4 values were significantly higher than female as in Milinković *et al.* (*10*). Free T4 values were partitioned by age in Płaczkowska *et al*. study with a smallest LRL of 10.8 and greatest URL of 23.5 pmol/L (*1*). In our study fT4 and fT3 RIs significantly differed by gender and age. There were also differences in study designs in two major subjects: first was the selection of patients according to different cut-offs for anti-TPO levels. In Inal *et al.* study National Academy of Clinical Biochemistry (NACB) guidelines criteria was applied and any patient having detectable anti-TPO was excluded from the study, thus in this study the URLs of TSH were quite low, and also in Friis-Hansen and Hilsted (*5*, *18*, *24*). Anti-TPO positive subjects were excluded and TSH URLs decreased after exclusion. In our study we excluded both anti-TPO and anti-Tg positive results and our URL was similar to that of Friis-Hansen and Hilsted (*18*). Hollowell *et al.* also mentioned about dependency of TSH results on anti-TPO levels (*25*). Another point is the thyroid ultrasonography (TUS) evaluation for the selection of reference individuals. In our study and other indirect studies this was not possible, however, TUS is not recommended in even strict NACB guidelines since it is not proven to be associated with TSH RIs in some studies (*7*). However, if patient selection could be made together with TUS results, it would contribute to the selection of reference individuals; so this may be the considered as the weakness of the study.

**Table 4:** Studies about reference intervals of thyroid hormones

**Reference / N**	**Statistical approach**	**Technology / device**	**TSH (mIU/L)**	**fT4 (pmol/L)**	**fT3 (pmol/L)**
(1) TSH: 105,927, fT4: 41,400	Indirect, Hoffman, RCV	CLIA/ Siemens	0.39-5.20	LRL = 10.9 URL = 23.5	–
(7) 272	Direct, nonparametric percentile, t-test, ANOVA	ECLIA/ Roche	0.56-4.45	–	–
(26) 146,801	Indirect, nonparametric percentile, multivariate regression	CLIA/ Beckman Unicel DXI	0.362-5.280	–	–
(8) 250	Direct, nonparametric percentile, student-t test, ANOVA	ECLIA/ Roche E170	0.75-5.32	12.29-20.03	4.11-6.32
(9) 2124	Direct, Parametric / Kruskal Wallis, Mann whitney U	CLIA/ Abbott Architect	M: 0.47-2.84 F: 0.47-3.08	–	–
(10) 22,860	Indirect, nonparametric percentile, Z statistics	CLIA/ Abbott Architect	M: 0.91-4.01 F: 0.58-4.20	M:10.8-18.3 F:11.5-15.4	–
(5) TSH: 55,318 fT4: 62,713	Indirect, nonparametric percentile, Z statistics	ECLIA/ Roche Elecsys	0.43-3.93	11.98-21.33	–
(21) 217	Direct, nonparametric percentile	CLIA/ Abbott Architect	0.17-4.23	11.24-26.86	2.56-6.36
(18) 489	Direct, parametric percentile	ECLIA/ Roche Modular E170	0.64-4.7	–	–
(22) 742	Direct, *a posteriori*, ANOVA	CLIA/ Architect i2000	0.30-4.32	9.8-18.6	–
F – female. M – male. N - number of patients. RCV – reference change value. CLIA - chemiluminescence immunoassay. ECLIA - electrochemiluminescence immunoassay. TSH - thyroid stimulating hormone. fT4 - free thyroxine. fT3 - free triiodothyronine.					

Another important difference is the appliance of different statistical procedures and interpretation of statistical significance. In case of laboratory results, a statistically significant difference does not mean a clinical significance all the time. Biological variations and/or RCVs are now the important criteria of effect size to test the clinical significance (*26*). In our study, we used standard deviation Z test to compare the subgroups, and RCVs to compare our RIs with those of the manufacturer. We saw that manufacturer RIs should be tested before applying it. Another important point is that the percent difference between LRLs for TSH was 51.2%, a difference smaller than RCV. But when we use the manufacturer provided interval of 0.27-4.20 mIU/L, patients having low TSH values < 0.41 mIU/L seems to be misdiagnosed as normal. The important question, is a 51.2% difference of TSH LRL, clinically significant? Thus, apart from establishing accurate RIs, the clinician should be informed about the RCV of the analyte to decide about any change in patient’s status in two consecutive measurements. This approach may soon replace the classic RI assessment. For many analytes like TSH, CV_İ_ is far more smaller than CV_G_. For such analytes, two consecutive results from a subject may be within the population-based RI but may not necessarily indicate a normal thyroid function (*27*).

In our study TSH values decreased with age, a pattern showing iodine deficiency. There are several studies in literature confirming this relationship (*28*, *29*). Maintenance of an iodine deficiency programme improved this deficiency status of Turkish people, remarkably at city centers (*30*). In a recent study in 2014, Istanbul was stil found mildly iodine deficient (*31*). According to Van de Ven *et al.,* an inverse relationship between TSH and age is usually observed in populations with a history of iodine deficiency (*32*). From a pathophysiological point of view, a chronic mild to moderate iodine deficiency makes chronic TSH stimulation causing functional thyroid autonomy. This situation in the elderly is a long-term index reflecting mild to moderate iodine deficiency lasting decades, more than actual iodine status. Reference interval width (RIW) is a useful measure to assess the different impact on normal values. In our study we also observed that TSH RIW values of age subgroups got wider progressively by aging; this may be another index of long-term iodine deficiency in Turkish population.

Indirect method is a satisfactory and recommended way of establishing population based RIs, with a large set of data covering the variability of the population. Differences should be compared with RCVs to decide whether they are clinically significant or not. Besides RIs, laboratories should inform clinicians about RCV of analytes for a better interpretation.
